# Exposure to proton pump inhibitors is associated with the development of pediatric autoimmune diseases

**DOI:** 10.3389/fped.2023.1157547

**Published:** 2023-03-27

**Authors:** Laura Räisänen, Heli Viljakainen, Kaija-Leena Kolho

**Affiliations:** ^1^ Department of Pediatrics, Tampere University Hospital, Tampere, Finland; ^2^ Public Health Research Program, Folkhälsan Research Center, Helsinki, Finland; ^3^ Children’s Hospital, University of Helsinki and HUS, Helsinki, Finland; ^4^ Faculty of Medicine and Medical Technology, Tampere University, Tampere, Finland

**Keywords:** autoimmune thyroiditis, inflammatory bowel diseases, juvenile idiopathic arthritis, omeprazole, type 1 diabetes mellitus

## Abstract

Proton pump inhibitors (PPIs) have been associated with decreased gut microbiota diversity. Disrupted gut microbiota composition has been reported in several autoimmune diseases (ADs), such as type 1 diabetes mellitus (DM), autoimmune thyroiditis (AIT), juvenile idiopathic arthritis (JIA), and inflammatory bowel diseases (IBD). We investigated whether PPIs are associated with the development of ADs in children and concluded that PPI exposures could be related to the onset of ADs, especially IBD and potentially AIT as well.

## 1. Introduction

Autoimmune diseases (ADs) are a group of complex immunological disorders, characterized by inflammation due to loss of tolerance to self-antigens. This study focused on four common pediatric ADs with especially high prevalence in Finland: type 1 diabetes (DM), autoimmune thyroiditis (AIT), juvenile idiopathic arthritis (JIA), and inflammatory bowel diseases (IBD). These ADs were selected to represent pediatric autoimmunity in general, because despite different disease outcomes, the pathogenesis of these ADs may resemble each other, including the presence of T-cell organ infiltrations (1–5). Most T-cells reside in the gut, which is the largest immunological organ in the body with numerous antigen-immune system contacts (6). After penetrating the gut mucosal barrier, antigens are introduced by the antigen presenting cells to the T-cells (7). If the gut mucosal barrier is compromised, for instance due to disrupted gut microbiota composition, an influx of antigens and excess stimulation of the immune system may take place (8). This phenomenon could contribute to the onset of any of these four ADs by stimulating different types of autoantibodies (9–12).

The incidence of ADs are increasing worldwide, and the incidence of pediatric DM, AIT, JIA, and IBD in Finland are among the highest in the world (13, 14). Since genetic factors are relatively stable, the potential roles of environmental factors behind the onset of ADs have been studied vigorously (15). Environmental factors may interact with gut microbiota, and disruption of gut microbiota composition has been demonstrated in several ADs (16–20).

In children, proton pump inhibitors (PPIs) are used to lower stomach acidity in several gastroesophageal problems such as short-term treatment of symptomatic gastro-esophageal reflux disease (GERD), erosive esophagitis healing, peptic ulcer disease therapy, Helicobacter pylori eradication, and pediatric eosinophilic esophagitis—and their prescriptions have been increasing within the past years (21). Sadly, over half of PPIs prescriptions are off-label, for instance for treating chronic cough, as stress ulcer prophylaxis, to prevent upper gastrointestinal bleeding for patients in intensive care units, etc (22). Even though PPIs are generally regarded safe, in children they have been shown to increase the risk of enteric infection and bone fractures (when used before the age of 1 year), and to have association with development of allergic disorders (22). These findings suggest that PPIs may have an influence on the immune system.

Long-term reduction of gastric acid secretion after PPI use may increase the risk of imbalance in the gut microbiota composition (23, 24). This disrupted homeostasis of gut microbiota composition may distract the production of gut microbial metabolites, which are required to maintain a well-functioning gut barrier, leading to impaired gut barrier function (25). Sequentially, breached gut barrier would introduce antigens in the intestines to the immune system, which may stimulate autoimmune responses (26). In addition, PPI use has been associated with an increased risk for microbial load and *Clostridium difficile* infection (27), which may promote inflammatory response due to intestinal proinflammatory release (28). Intriguingly, despite increasing frequency of both PPI prescriptions and ADs, and despite suggested connection between PPIs and autoimmune responses, studies associating PPIs and pediatric ADs have been scarce. This study aims to investigate whether the use of PPIs in childhood is associated with the development of pediatric onset ADs in general (represented by DM, AIT, JIA, and IBD).

## 2. Materials and methods

The study population for this matched case-control study was derived from the Finnish Health in Teens (Fin-HIT) cohort—a nationwide school-based cohort to investigate health and health-related behaviors of Finnish children. The cohort was established in 2011–2014, mostly through school recruitment. It comprised over 11,000 children (born 2000–2005) from densely populated areas across Finland without specific exclusion criteria. More details on the cohort has been described elsewhere (29).

Based on unique personal identity code of every Finnish resident, children in the Fin-HIT cohort were linked to: (1) the Special Reimbursement Register (SRR)—containing date of diagnosis and specialist-verified diagnosis of patients with chronic diseases, who are entitled to drug refunds regardless of their socioeconomic status; and (2) the Drug Purchase Register (DPR)—containing dispensation dates and Anatomical Therapeutic Chemical (ATC) codes of all prescription-based drug purchases in Finland. These national registers are maintained by the Finnish Social Insurance Institution and their excellent coverage and reliability has been described previously (30, 31).

The outcome of this study was a primary autoimmune diagnosis by the end of the follow-up in 31 December 2018—when the median age of the participants was 16 years. The ICD-10 codes [International Classification of Diseases (ICD), 10th revision] were used to identify patients with DM (E10), JIA (M08), and IBD (including Crohn's disease (K50), ulcerative colitis and IBD unclassified (K51)) from the SRR. Regarding AIT, patients were identified from the DPR using the ATC code H03AA01 for thyroxin (a prescription-only drug used for AIT), because not everyone using low-priced thyroxin is applying for special reimbursement. Of the over 11,000 children in the Fin-HIT cohort, 242 developed a primary AD after the first year of life and were included in this study as the case group as previously described (32). Each child in the case group was matched with four to ten children without studied ADs from the same cohort, with similar age (0 to 30 days of differences in age), sex, and residential area. These children (*N* = 2,147) were marked as matched controls. In total, the study population comprised 2,389 children from the Fin-HIT cohort.

Data on PPI purchases were obtained from the DPR using ATC codes starting with A02B. Different types of PPIs purchases in this study were: omeprazole (A02BC01), pantoprazole (A02BC02), lansoprazole (A02BC03), rabeprazole (A02BC04), and esomeprazole (A02BC05). These PPIs were analyzed as one group. The data were collected from birth until the index date—i.e., 6 months before the date of diagnosis in cases/ respective date in matched controls. PPI purchases 6 months prior to diagnoses were excluded from the analysis to limit the possibility that these PPIs were used during exacerbation phase of ADs. This time window was chosen because previous studies have reported median diagnostic delays for JIA and IBD as ranging from 3 to 5 months (33, 34). Children with registered PPI purchases were categorized as “exposed” (regardless of the number of purchases) and those without registered PPI purchases were categorized as “not exposed”. The potential relationship between pre-diagnostic PPI exposures and the development ADs as a group was investigated.

Potential confounders, such as antibiotic purchases before the age of three years (using ATC code starting with J01) and systemic cortisone purchases before diagnosis (using ATC codes starting with H02) were considered in the analysis. The background data of the study population are presented as mean and standard deviation (SD), median (interquartile range, IQR) or number/proportion (%). Before index date, PPI exposures of each case were compared with the exposures of his/her matched controls, and their relationship with the development of ADs was estimated using conditional logistic regression with strata analysis (35). Results were presented with Odds Ratio (OR) and 95% confidence interval (CI). The software used was IBM SPSS Statistics 26.0 and a 5% statistical significance level was adopted.

## 3. Results

The background characteristics of the 242 children who developed ADs (cases) and their 2,147 matched controls are presented in Table 1. In this study population of 2,389 children, 46 children (1.9%) were exposed to PPIs (comprising a total of 95 purchases). Only 11 children had repeated PPI purchases, of which 3 developed an AD (Table 2). In the case group, 9 children (3.7%) were exposed to PPIs (comprising a total of 26 purchases). Omeprazole was the most common type of PPI purchase before the index date (71.0% of all PPI purchases).

**Table 1:** Background characteristics of children in the study population.

	DM (*N* = 101)	AIT (*N* = 63)	JIA (*N* = 52)	IBD (*N* = 26)	Cases^a^ (*N* = 242)	Matched controls^b^ (*N* = 2,147)
Age at the end of follow-up (years), mean ± SD	16.5 ± 1.6	17.1 ± 1.1	16.7 ± 1.3	16.7 ± 1.2	16.7 ± 1.4	16.7 ± 1.4
Sex, *n* (%)						
Girl	41 (40.6)	44 (69.8)	42 (80.8	13 (50.0)	140 (57.9)	1,222 (56.9)
Boy	60 (59.4)	19 (30.2)	10 (19.2)	13 (50.0)	102 (42.1)	925 (43.1)
Residential area, *n* (%)						
Capital (South)	33 (32.7)	18 (28.6)	10 (19.2)	10 (38.5)	71 (29.3)	635 (29.6)
Inner South	6 (5.9)	7 (11.1)	13 (25.0)	4 (15.4)	30 (12.4)	262 (12.2)
West	9 (8.9)	16 (25.4)	10 (19.2)	2 (7.7)	37 (15.3)	287 (13.4)
East	33 (32.7)	16 (25.4)	9 (17.3)	5 (19.2)	63 (26.0)	569 (26.5)
North	20 (19.8)	6 (9.5)	10 (19.2)	5 (19.2)	41 (16.9)	394 (18.4)
**Median age of diagnosis, years (IQR)**	8.7 (4.8–12.0)	14.1 (10.0–16.1)	10.3 (4.4–13.1)	11.8 (9.5–13.6)	11.0 (6.0–13.8)	

^a^ Cases = children with autoimmune diseases (represented with DM (type 1 diabetes mellitus), AIT (autoimmune thyroiditis), JIA (juvenile idiopathic arthritis), and IBD (inflammatory bowel diseases)).

^b^ Each child in the case group were matched with four to ten children with similar age, sex, and residential area.

**Table 2:** Proton pump inhibitor (PPI) exposures in the matched case-control study population.

	DM (*N* = 101)	AIT (*N* = 63)	JIA (*N* = 52)	IBD (*N* = 26)	Cases^a^ (*N* = 242)	Matched controls^b^ (*N* = 2,147)
Number of children exposed to PPIs, *n* (%)	1 (1.0)	4 (6.3)	2 (3.8)	2 (7.7)	9 (3.7)	37 (1.7)
Boys	1 (1.0)	2 (3.2)	0	1 (3.8)	4 (1.7)	14 (0.6)
Girls	0	2 (3.2)	2 (3.8)	1 (3.8)	5 (2.1)	23 (1.1)
Number of children with more than one PPI purchase, *n* (%)	1 (1.0)	1 (1.6)	0	1 (3.8)	3 (1.2)	8 (0.4)
Range of PPI purchases	0–2	0–10	0–1	0–8	0–10	0–11
Total number of PPI purchases	2	13	2	9	26	69
Number of children exposed to PPIs within 2 years before index date	1 (1.0)	1 (1.6)	2 (3.8)	2 (7.7)	6 (2.5)	10 (0.5)

^a^ Cases = children with autoimmune diseases (represented with DM (type 1 diabetes mellitus), AIT (autoimmune thyroiditis), JIA (juvenile idiopathic arthritis), and IBD (inflammatory bowel diseases)).

^b^ Each child in the case group were matched with four to ten children with similar age, sex, and residential area.

**Figure 1:**
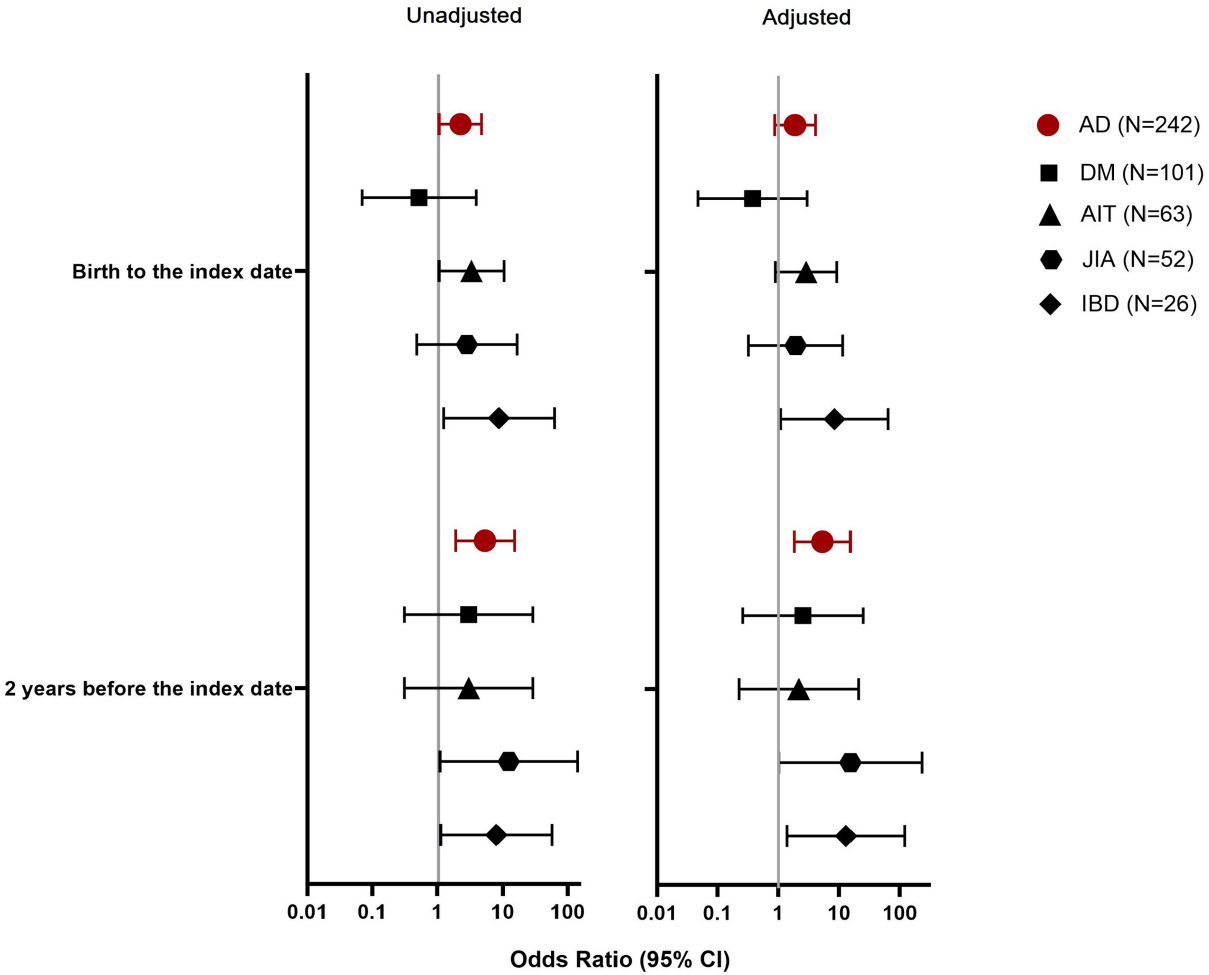
Association between proton pump inhibitor (PPI) exposure in different observation periods and the development of an autoimmune disease (AD), represented by type 1 diabetes (DM), autoimmune thyroiditis (AIT), juvenile idiopathic arthritis (JIA), or inflammatory bowel diseases (IBD).

PPI exposures from birth to the index date were related with the development of ADs (OR 2.25, 95% CI, 1.06–4.78), especially IBD (OR 8.81, 95% CI, 1.23–63.3) and AIT (OR 3.34, 95% CI, 1.06–10.5) (Figure 1). Of the PPI purchases in the case group, 67% occurred within 2 years before diagnosis and PPI exposures in this period showed an even stronger association with the development of an AD (OR 5.40, 95% CI, 1.90–15.3).

Children who were exposed to PPIs had more frequent antibiotic purchases before the age of 3 years compared to children who were not exposed to PPIs (Table 3). As early antibiotic exposures may associate with the development of ADs (32, 36, 37), we adjusted the PPI analyses with early life antibiotic exposures as a potential confounder. After this adjustment, the association between PPI exposures from birth to the index date and the onset of ADs was diminished (OR 1.88, 95% CI, 0.87–4.07). This was also seen regarding AIT (OR 2.86, 95% CI, 0.89–9.16), but not regarding IBD (OR 8.38, 95% CI, 1.09–64.4). However, the association between PPIs purchased within 2 years before index date and ADs was retained (OR 5.27, 95% CI, 1.81–15.3), especially regarding IBD (OR 13.0, 95% CI, 1.39–121).

**Table 3:** Background characteristics of children with and without exposures to proton pump inhibitors (PPIs).

	Children exposed to PPIs (*N* = 46)	Children without PPI exposures (*N* = 2,343)	*p*-value^a^
**Sex, *n* (%)**			0.594
Boys	18 (0.8)	1,009 (42.2)	
Girls	28 (1.2)	1,334 (55.8)	
**Maternal socioeconomic status, *n* (%)**			0.949
Upper-level employees	17 (37.0)	698 (29.8)	
Lower-level employees	15 (32.6)	922 (39.4)	
Manual workers, students, self-employed, housewife, others (unemployed, pensioners)	12 (26.1)	676 (28.9)	
Missing	2 (4.4)	47 (2.0)	
**Preterm birth, *n* (%)**			0.112
Yes	0	124 (5.3)	
No	45 (97.8)	2,209 (94.3)	
Missing	1 (2.2)	10 (0.4)	
**Antibiotic exposures before the age of 3 years^b^, *n* (%)**			<0.001
None	2 (4.3)	310 (13.2)	
1–2 times	10 (21.7)	932 (39.8)	
3–6 times	11 (23.9)	539 (23.0)	
More than 6 times	23 (50.0)	562 (24.0)	

^a^ Pearson's chi square.

^b^ All types of antibiotic exposures, used categories were based on interquartile range (IQR).

No differences were found regarding sex, preterm birth, nor maternal socioeconomic status when the background of PPI-exposed and non-exposed children were compared. Of the PPIs purchased within 6–24 months before AD diagnosis, only two PPIs were purchased close to a systemic glucocorticoid purchase (one was purchased at the same time, and one was purchased three months before glucocorticoid). In both times, these PPIs were purchased at approximately one year before JIA diagnosis. Furthermore, only 10 children in this study were exposed to H2-antagonists, thus no further analysis on this medication were performed.

## 4. Discussion

Our study is the first pilot study to estimate the relationship between exposure to PPIs and the onset of common pediatric ADs (DM, AIT, JIA, or IBD) in a mutual setting. We concluded that PPI exposures may associate with the development of ADs, especially when used within 2 years before diagnosis. However, this might be explained by more prominent relationship of PPI exposures with IBD.

In general, PPI purchases were relatively low in our study population. Yet, we found an association between PPI exposures and the onset of ADs, especially IBD and potentially AIT. Our results correspond with a previous pediatric study associating PPI and IBD (38), and with two distinct cohort studies in adults, presenting an increased risk of AIT and IBD after exposures to PPIs (39, 40). No solid evidence on mechanisms linking PPI and ADs have been reported, but this could involve disruption of gut microbiota. Long-term PPI use has been shown to influence the balance of gut microbiota composition (23, 24), and disrupted gut microbiota composition has been reported in for instance both IBD and AIT (19, 41). Altered gut microbiota has also been described in DM and JIA (42, 43), but we observed no connection between PPI exposures and the onset of these diseases, most likely due to the low prevalence of pre-diagnostic PPI purchases.

PPIs have been used to treat several gastrointestinal problems due to their ability to inhibit gastric acid secretion by antagonizing the H+/K+ ATPase pump in the parietal cells (44). However, reduced gastric acidity may interrupt protein digestion, because pepsin activity in the stomach dependents on an acidic environment. Sequentially, some inappropriately digested peptides may act as epitopes for intestinal immune cells and induce unwanted immune responses. This may explain why in our study more recent PPI exposures showed high odds of developing an AD.

Finally, omeprazole—the most commonly used PPI in this study—has been shown to interfere with the reactive oxygen species (ROS) production capacity of neutrophils (45). Since ROS is needed in a resolution process of inflammation (46), a defect in this function may lead to disrupted gut epithelial barrier and structural damages of the intestines as seen in IBD. In addition, ROS are necessary in suppression of T-cells. Therefore, lack of this suppression may contribute to local inflammations and autoimmune reactions (47). Hence, the link between recent PPI exposures, neutrophil's ROS production, and the onset of ADs require further studies.

The strength of this study lies in the reliability and coverage of longitudinal data from national registers (31). In Finland PPIs could not be purchased for children without prescription. Therefore, our register-based data covers all PPI purchases of our study population. Furthermore, we studied several ADs in a mutual setting, using a comprehensive Fin-HIT cohort with small variations in socioeconomic status as the source of study population (14). In addition, the controls were matched for age, sex, and residential area to further limit the number of potential confounding factors. We also considered several possible confounding background factors of children with and without exposure to PPIs and did not find significant differences other than early antibiotic use. Yet, after adjusting our analysis with early childhood antibiotic use, our conclusion did not change. In adults, PPIs are often used when systemic glucocorticoids are used for treating ADs, for example. We investigated this potential protopathic bias and discovered that in our data, the pre-diagnostic systemic glucocorticoid purchase was extremely rare. By using this type of study setting, we were able to investigate the relationship between PPI exposures and the development of the four pediatric ADs together as one group, and to reliably compare each of these diseases to one another.

Regarding limitations, we have no information on the children's genetic susceptibility to ADs, the duration of PPI exposures, nor why the PPIs were used in the first place. Moreover, we could not guarantee whether the purchased PPIs were used or not. However, since monitoring actual PPI consumption of more than 11,000 children for over a decade is not possible, we chose the second-best study design based on nationally registered PPI purchases. In Finland, adults can purchase small amounts of PPIs without prescriptions. In theory, these PPIs could be given to children and would not be registered in our data. Nevertheless, in our experience this happens very seldomly in Finland, since parents would usually rely on medical advice and prescriptions regarding the wellbeing of their children. Since PPI exposures in the Fin-HIT cohort were relatively scarce, this may decrease the statistical power of our study during analysis. Therefore, our study should be considered as a pilot study for future research directions.

In conclusion, exposure to PPIs may increase the likelihood of developing ADs, especially IBD. More studies involving higher volume of PPI exposures to confirm the relationship between PPI exposures and the development of ADs are warranted.

## Data Availability

The raw data supporting the conclusions of this article will be made available by the principal investigator upon request.

## References

[1] Paschou SA Papadopoulou-Marketou N Chrousos G Kanaka-Gantenbein C. On type 1 diabetes mellitus pathogenesis. Endocr Connect. (2017) 7(1):R38–R46. 10.1530/EC-17-0347 29191919 PMC5776665

[2] Pugliese A. Autoreactive T cells in type 1 diabetes. J Clin Investig. (2017) 127(8):2881–91. 10.1172/JCI94549 28762987 PMC5531393

[3] Antonelli A Ferrari SM Corrado A Di Domenicantonio A Fallahi P. Autoimmune thyroid disorders. Autoimmun Rev. (2014) 14(2):174–80. 10.1016/j.autrev.2014.10.016 25461470

[4] Prakken B Albani S Martini A. Juvenile idiopathic arthritis. Lancet. (2011) 377:2238–49. 10.1016/S0140-6736(11)60244-4 21684384

[5] Giuffrida P Corazza GR Di Sabatino A. Old and new lymphocyte players in inflammatory bowel disease. Dig Dis Sci. (2017) 63(2):277–88. 10.1007/s10620-017-4892-4 29275447

[6] van Wijk F Cheroutre H. Mucosal T cells in gut homeostasis and inflammation. Expert Rev Clin Immunol. (2010) 6(4):559–66. 10.1586/eci.10.34 20594129 PMC2976609

[7] Mann ER. Intestinal antigen-presenting cells in mucosal immune homeostasis: crosstalk between dendritic cells, macrophages and B-cells. WJG. (2014) 20(29):9653. 10.3748/wjg.v20.i29.9653 25110405 PMC4123356

[8] Paone P Cani PD. Mucus barrier, mucins and gut microbiota: the expected slimy partners? Gut. (2020) 69(12):2232–43. 10.1136/gutjnl-2020-322260 32917747 PMC7677487

[9] Taplin C Barker J. Autoantibodies in type 1 diabetes. Autoimmunity. (2008) 41(1):11–8. 10.1080/08916930701619169 18176860

[10] Fröhlich E Wahl R. Thyroid autoimmunity: role of anti-thyroid antibodies in thyroid and extra-thyroidal diseases. Front Immunol. (2017) 8:521. 10.3389/fimmu.2017.00521 28536577 PMC5422478

[11] Mahmud SA Binstadt BA. Autoantibodies in the pathogenesis, diagnosis, and prognosis of juvenile idiopathic arthritis. Front Immunol. (2019) 9:3168. 10.3389/fimmu.2018.03168 30693002 PMC6339949

[12] Mitsuyama K. Antibody markers in the diagnosis of inflammatory bowel disease. WJG. (2016) 22(3):1304. 10.3748/wjg.v22.i3.1304 26811667 PMC4716040

[13] Lerner A Jeremias P Matthias T. The world incidence and prevalence of autoimmune diseases is increasing. Int J Celiac Dis. (2016) 3(4):151–5. 10.12691/ijcd-3-4-8

[14] Räisänen L Viljakainen H Sarkkola C Kolho KL. Perinatal risk factors for pediatric onset type 1 diabetes, autoimmune thyroiditis, juvenile idiopathic arthritis, and inflammatory bowel diseases. Eur J Pediatr. (2021) 180(7):2115–23. 10.1007/s00431-021-03987-3 33624160 PMC8195774

[15] Vojdani A Pollard KM Campbell AW. Environmental triggers and autoimmunity. Autoimmune Dis. (2014) 2014:798029–2. 10.1155/2014/798029 25610638 PMC4290643

[16] Han H Li Y Fang J Liu G Yin J Li T Gut Microbiota and type 1 diabetes. Int J Mol Sci. (2018) 19(4):995. 10.3390/ijms19040995 29584630 PMC5979537

[17] Virili C Fallahi P Antonelli A Benvenga S Centanni M. Gut microbiota and Hashimoto’s thyroiditis. Rev Endocr Metab Disord. (2018) 19(4):293–300. 10.1007/s11154-018-9467-y 30294759

[18] Arvonen M Berntson L Pokka T Karttunen TJ Vähäsalo P Stoll ML. Gut microbiota-host interactions and juvenile idiopathic arthritis. Pediatr Rheumatol Online J. (2016) 14(1):44. 10.1186/s12969-016-0104-6 27448997 PMC4957868

[19] Kolho KL Korpela K Jaakkola T Pichai MVA Zoetendal EG Salonen A Fecal microbiota in pediatric inflammatory bowel disease and its relation to inflammation. Am J Gastroenterol. (2015) 110(6):921–30. 10.1038/ajg.2015.149 25986361

[20] Comito D Romano C. Dysbiosis in the pathogenesis of pediatric inflammatory bowel diseases. Int J Inflamm. (2012)2012:687143–7. 10.1155/2012/687143 PMC3363416 22685684

[21] Levy EI Salvatore S Vandenplas Y de Winter JP. Prescription of acid inhibitors in infants: an addiction hard to break. Eur J Pediatr. (2020) 179(12):1957–61. 10.1007/s00431-020-03855-6 33150519

[22] Dipasquale V Cicala G Spina E Romano C. A narrative review on efficacy and safety of proton pump inhibitors in children. Front Pharmacol. (2022) 13:839972. 10.3389/fphar.2022.839972 35222047 PMC8866943

[23] Imhann F Bonder MJ Vich Vila A Fu J Mujagic Z Vork L Proton pump inhibitors affect the gut microbiome. Gut. (2016) 65(5):740–8. 10.1136/gutjnl-2015-310376 26657899 PMC4853569

[24] Bruno G Zaccari P Rocco G Scalese G Panetta C Porowska B Proton pump inhibitors and dysbiosis: current knowledge and aspects to be clarified. WJG. (2019) 25(22):2706–19. 10.3748/wjg.v25.i22.2706 31235994 PMC6580352

[25] Ghosh S Whitley CS Haribabu B Jala VR. Regulation of intestinal barrier function by microbial metabolites. CMGH. (2021) 11(5):1463–82. 10.1016/j.jcmgh.2021.02.007 33610769 PMC8025057

[26] Mu Q Kirby J Reilly CM Luo XM. Leaky gut as a danger signal for autoimmune diseases. Front Immunol. (2017) 8:598. 10.3389/fimmu.2017.00598 28588585 PMC5440529

[27] Trifan A Stanciu C Girleanu I Stoica OC Singeap AM Maxim R Proton pump inhibitors therapy and risk of Clostridium difficile infection: systematic review and meta-analysis. WJG. (2017) 23(35):6500–15. 10.3748/wjg.v23.i35.6500 29085200 PMC5643276

[28] Solomon K. The host immune response to *Clostridium difficile* infection. Ther Adv Inf. (2013) 1(1):19–35. 10.1177/2049936112472173 PMC4040718 25165542

[29] de Oliveira Figueiredo RA Simola-Ström S Rounge TB Viljakainen H Eriksson JG Roos E Cohort profile: The Finnish Health in Teens (Fin-HIT) study: a population-based study. Int J Epidemiol. (2019) 48(1):23–24h. 10.1093/ije/dyy189 30212855 PMC6380305

[30] Niemelä H. Social security in Finland. Helsinki, Finland: Social Insurance Institution (KELA), Finnish Centre for Pensions (ETK), Finnish Pension Alliance (TELA), and Finnish Ministry of Social Affairs and Health (2006).

[31] Furu K Wettermark B Andersen M Martikainen JE Almarsdottir AB Sorensen HT. The nordic countries as a cohort for pharmacoepidemiological research. Basic Clin Pharmacol Toxicol. (2010) 106(2):86–94. 10.1111/j.1742-7843.2009.00494.x 19961477

[32] Räisänen LK Kääriäinen SE Sund R Engberg E Viljakainen HT Kolho KL. Antibiotic exposures and the development of pediatric autoimmune diseases: a register-based case–control study. Pediatr Res. (2022). 10.1038/s41390-022-02188-4. [Epub ahead of print] PMC10033398 35854091

[33] Aoust L Rossi-Semerano L Koné-Paut I Dusser P. Time to diagnosis in juvenile idiopathic arthritis: a French perspective. Orphanet J Rare Dis. (2017) 12(1):43. 10.1186/s13023-017-0586-4 28241879 PMC5329952

[34] Sulkanen E Repo M Huhtala H Hiltunen P Kurppa K. Impact of diagnostic delay to the clinical presentation and associated factors in pediatric inflammatory bowel disease: a retrospective study. BMC Gastroenterol. (2021) 21(1):364. 10.1186/s12876-021-01938-8 34620103 PMC8495911

[35] Rose S van der Laan MJ. Why match? Investigating matched case-control study designs with causal effect estimation. Int J Biostat. (2009) 5(1):Article 1. 10.2202/1557-4679.1127 20231866 PMC2827892

[36] Virta L Auvinen A Helenius H Huovinen P Kolho KL. Association of repeated exposure to antibiotics with the development of pediatric crohn’s disease–A nationwide, register-based Finnish case-control study. Am J Epidemiol. (2012) 175(8):775–84. 10.1093/aje/kwr400 22366379

[37] Horton DB Scott FI Haynes K Putt ME Rose CD Lewis JD Antibiotic exposure and juvenile idiopathic arthritis: a case-control study. Pediatrics (Evanston). (2015) 136(2):e333–43. 10.1542/peds.2015-0036 PMC4516942 26195533

[38] Schwartz NRM Hutfless S Herrinton LJ Amsden LB Fevrier HB Giefer M Proton pump inhibitors, H2 blocker use, and risk of inflammatory bowel disease in children. J Pediatr Pharmacol Therap. (2019) 24(6):489–96. 10.5863/1551-6776-24.6.489 31719810 PMC6836698

[39] Xia B Yang M Nguyen LH He Q Zhen J Yu Y Regular use of proton pump inhibitor and the risk of inflammatory bowel disease: pooled analysis of 3 prospective cohorts. Gastroenterology. (2021) 161(6):1842–52.e10. 10.1053/j.gastro.2021.08.005 34389338

[40] Lin SH Chang YS Lin TM Hu LF Hou TY Hsu HC Proton pump inhibitors increase the risk of autoimmune diseases: a nationwide cohort study. Front Immunol. (2021) 12:736036. 10.3389/fimmu.2021.736036 34659225 PMC8514990

[41] de FreitasCayres LC de Salis LVV Rodrigues GSP van Lengert AH Biondi APC Sargentini LDB Detection of alterations in the gut Microbiota and intestinal permeability in patients with hashimoto thyroiditis. Front Immunol. (2021) 12:579140. 10.3389/fimmu.2021.579140 33746942 PMC7973118

[42] Murri M Leiva I Gomez-Zumaquero JM Tinahones FJ Cardona F Soriguer F Gut microbiota in children with type 1 diabetes differs from that in healthy children: a case-control study. BMC Med. (2013) 11(1):46. 10.1186/1741-7015-11-46 23433344 PMC3621820

[43] van Dijkhuizen EHP Del Chierico F Malattia C Russo A Pires Marafon D ter Haar NM Microbiome analytics of the gut Microbiota in patients with juvenile idiopathic arthritis : a longitudinal observational cohort study. Arthritis Rheumatol (Hoboken, NJ). (2019) 71(6):1000–10. 10.1002/art.40827 PMC6593809 30592383

[44] Orel R Benninga MA Broekaert IJ Gottrand F Papadopoulou A Ribes-Koninckx C Drugs in focus: proton pump inhibitors. JPGN. (2021) 72(5):645–53. 10.1097/MPG.0000000000003063 33847286

[45] Zedtwitz-Liebenstein K Wenisch C Patruta S Parschalk B Daxböck F Graninger W. Omeprazole treatment diminishes intra- and extracellular neutrophil reactive oxygen production and bactericidal activity. Crit Care Med. (2002) 30(5):1118–22. 10.1097/00003246-200205000-00026 12006811

[46] Wéra O Lancellotti P Oury C. The dual role of neutrophils in inflammatory bowel diseases. J Clin Med. (2016) 5(12):118. 10.3390/jcm5120118 27999328 PMC5184791

[47] Arve-Butler S Mossberg A Schmidt T Welinder C Yan H Berthold E Neutrophils lose the capacity to suppress T cell proliferation upon migration towards inflamed joints in juvenile idiopathic arthritis. Front Immunol. (2022) 12:795260. 10.3389/fimmu.2021.795260 35095871 PMC8792960

